# Relationship between Angular Measurements and Facial Shape of Young Ivorians with Normal Dental Occlusion

**DOI:** 10.1155/2018/6395910

**Published:** 2018-02-25

**Authors:** Moussa Diomande, Jean-Bertin Beugre, Mariam Konaté Kady Koueita, Frédéric Vaysse

**Affiliations:** ^1^Laboratory of Biomorphology and Imaging, School of Dentistry, Université Félix Houphouët-Boigny, Abidjan, Côte d'Ivoire; ^2^Department of Paleoanthropology, Anthropological Sciences of Development Institute, University Félix Houphouët-Boigny, Abidjan, Côte d'Ivoire; ^3^Laboratory of Molecular Anthropology and Imaging of Synthesis, University Paul Sabatier, Toulouse, France

## Abstract

**Objective:**

The aim of this study was to perform an analysis of angular measurements (from both the full face and profile), according to shapes of the human face.

**Method:**

It was a descriptive and cross-sectional study of 108 black Ivorian subjects. For each subject selected, two standardized photographs (full face and profile) were taken, followed by anthropometric measurements. The data collected were analyzed using the SPSS 20.0 statistics software for Windows.

**Results:**

In the present work, the faces were considered according to three particular qualifiers: broad face, medium face, and narrow face. Thus, 45.37% of the faces in this study were large, 31.48% on average, and 23.15% narrow. The interlabial angles of average face and long face were wider than that of large face with *p* < 0.01. The angle of the facial width was higher for large face and average face, compared to narrow face (*p* < 0.001).

**Conclusion:**

Median and bilateral angles lead to rational understanding of the various shapes of the human face.

## 1. Introduction

From the time of Ancient Greece to that day, the human face has been subject to various analyses [1]. And the early authors who paid attention to that topic were artists such as Pythagora and Leonard De Vinci. They set new standards aiming to express the harmony of this part of the body [2, 3].

On top of the above stated, facial analyses have been widely used for the biological classification of individuals on the basis of their shape referred to as morphotype. The facial morphotype stands as the typical shape of a face [4]. It is usually determined thanks to anthropometrical measures of the face. These descriptive measures allowed organizing the face shapes in different forms [5]. We then refer to very large face, large face, average or round face, long face or narrow, and very long face, according to international standards [6, 7]. This organization allowed Yesmin et al. (2014) to analyze facial cues among Malaysians. Based on the measurement of the length and facial width, these authors arranged the studied faces according to these predetermined shapes [8]. These different shapes of face are affected by ethnical, ecological, biological, geographical, gender, age, and nutritional factors [9–11]. Over our lifespan, for instance, the facial architecture grows through a development (formation) and senescence process. This change may be studied through angular measures [12]. Face shapes are also used to analyze some abnormalities. For example, the large face is sensitive to obstructive sleep apnea. And the symptoms feature a deep nasal breathing [13]. Moreover, other authors such as Cohen et al. (1994) and Oladipo et al. (2010) have shown that people with Apert syndrome are both hyperbrachycephalic and narrow-faced [14, 15].

In view of the above, the analysis of the morphological variability of the face reveals enormous interest in human biology, anthropology, medicine, and so forth. As well dependent on the measurement of the facial width and length, this variability of the morphology of the face could be explained through other geometric representations such as angular measurements. Clearly on the face there is a correlation between the facial shape and the angular measurements determined in the frontal and lateral direction. Despite the diversity of studies on the orofacial sphere, no study has sufficiently emphasized angular measures as factors that can influence the facial shape.

The aim of the this study was to analyze the correlation between the facial shape and the angular measures among Black Ivorians.

## 2. Materials and Methods

### 2.1. Sample

This was a cross-sectional study of 108 Black Ivorian subjects (53 female, 55 male). It was conducted with the approval and consent of all the participants. The subjects were aged 18–25 years (mean 20.5 years), and all had normal dental occlusion (Angle Class I).

To be included in the study, the subjects had to be natives of the Republic of Côte d'Ivoire, be free from any pathological disorders of the craniofacial soft tissues (bruising, ulceration, etc.), and have no severe craniofacial abnormalities. Subjects who had severe craniofacial antecedents or trauma, or who had undergone orthodontic or prosthetic treatment or orthognathic or other plastic surgery, were not included in the study.

### 2.2. Method

Two standardized photographs, one full face and one profile, were taken of each subject selected according to the above inclusion criteria. All the photographs were taken with the same digital camera (Nikon Coolpix, with a resolution of 5.2 megapixels, 3x optical zoom, and 40 mm macro focusing), mounted on a tripod, the height of which was adjusted so that the optical lens axis was always horizontal and the image sensor plane vertical. The height of the tripod was therefore adjusted to that of each subject for each photograph.

On the wall in front of each subject, a rectangular mirror measuring 100 × 50 cm was hung and fixed 40 cm from a horizontal line marked on the ground. While serving as a reference scale, measuring tape, the vertical and the horizontal, has provided the framework for taking the pictures. This framework has helped achieve true vertical and actual facial clues measurements.

The subject stood straight, feet together straddling the line, 100 mm from the camera, so that the subject's head and the camera are at the same height.

The photographic method described by Ferrario et al. [16] was used: when the photograph was being taken, subjects were to look straight ahead at the reflection of their pupils in the mirror in front of them (eyes levelled horizontal, and midline of face truly vertical). The subjects were asked to relax, with lips in resting position and hands hanging freely on each side of their body. The photographs were thus taken with the head in a natural posture. That is to say the position of the head must respect the Frankfort plan and be horizontal.

Blurred photographs and any with shadow images and/or contractions of facial muscles (e.g., creased or flattened chin pad) were discarded. All the subjects gave their consent after being informed of the study objectives.

**Figure 1:**
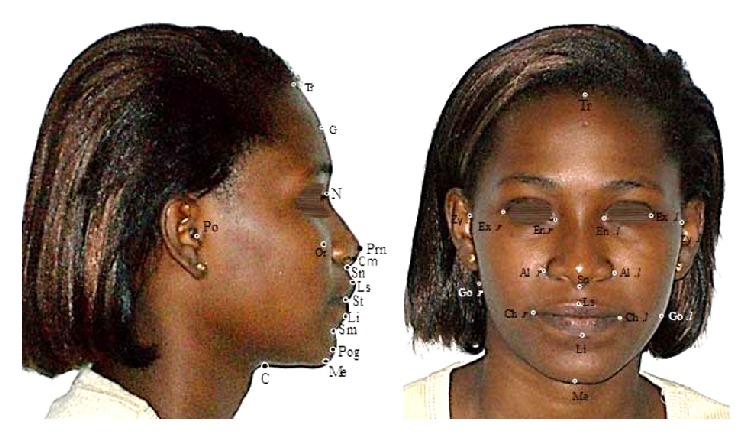
Landmarks used for profile photogrammetry.

The images obtained were printed on white sheets using one printer (HP Deskjet 3050). Landmarks and lines were then drawn by hand on these printouts by one operator using a graduated ruler and a protractor on the full face and profile views. The landmarks are shown in Figure 1.

**Figure 2:**
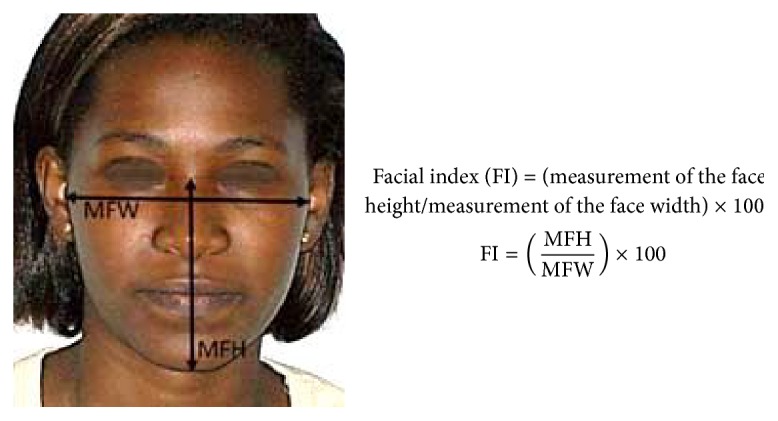
Face index determination method.

These landmarks were used to determine the face index according to the method adopted by Olivier (1960) and quoted by Yesmin et al. (2014) [8]. The facial index was calculated by dividing the measure of the distance between the nasion (N) and pogonion (Pog) points (that represent the facial height) by the measure of the interzygomatic distance (i.e., the facial width), multiplied by 100. It is literally presented as follows: facial index (FI) = (height of the face/facial width) × 100 (Figure 2).

Faces arranged according to the different forms were subject to some 20 angular measurements (among which, there were 8 full faces and 12 profiles).

The data collected were analyzed using the statistical software IBM SPSS 20.0 for Windows. The quantitative variables had a normal distribution as shown by their means and a Levene test. An univariate ANOVA was carried out to compare the photogrammetric variables according to appraisal category and gender (Table 4). The significance threshold was set at *p* < 0.05. The attempt to reduce alpha error accumulation was made with a Bonferroni test. When the null hypothesis was rejected, a Bonferroni test was used to correct errors and determine the level of differentiation between these subjects.

A reproducibility test was carried out on 20 subjects chosen randomly. On these 20 subjects, the same measurements were made again by the same operator two weeks later, and the first and second measurements were compared. Method error was calculated using the formula of Dahlberg [17], ME = √∑*d*^2^/2*n*, where *d* is the difference between the first and second measurements and *n* is the number of persons chosen randomly (Table 3).

## 3. Results

Discrepancies were 1.5° at most. On the basis of a *t*-test, comparison between the first measurements and the repeated ones has shown no significant difference. Hence the drawing method used was reliable enough as regards the clear identification of landmarks.

According to the distribution of the face indexes, a total of 45.37% (30 females, 19 males) of the subjects had a large face; 31.4814% (13 females, 21 males) had average face; and 23.15% (11 females, 14 males) had a long or narrow face.

Long and average face interlabial angles (Sn-Ls/Li-Sn) were wider than that of large face (*∗∗*), with *p* > 0.01. The angle of the face width (Zy.r-Tr-Zy.l) was higher for large face and average face compared to long face (*p* < 0.001). With *p* < 0.004, large face and average face (*∗*) had zygomatic angles Tr-Zy-Go (.*r*;* l)* inferior to that of long face (*∗∗*). In addition, the long face had an oral angle (Ch.*r*-Gn-Ch.*l*) width less than that of the others (*p* < 0.001).

Li/Li-Sn, Tr-Zy-Go (r, l), Zy.r-Tr-Zy.l, and Go.*r*-Gn-Go.*l* angles were significantly correlated to the shape of the face (with *p* < 0.01; *p* < 0.005; *p* < 0.00; and *p* < 0.04).

The angles Sn-Li/Li-Sn, Tr-Zy-Go (r, l or 1, 2), and Go.*r*-Gn-Go.*l* were negatively correlated to the index values of the face. This relationship was considerably higher regarding the zygomatic angles (Tr-Zy-Go 1.2) with a −0.8674-correlation coefficient. The angle Zy.r-Tr-Zy.l was positively correlated to the indexes values of the face with a 0.69 correlation coefficient.

On the ordinate are the angular measurements Sn-Ls/Li-Sm, Tr-Zy-Go (r, l), Go.*r*-Gn-Go.*l*; on the abscissa are the index values of the face. Depending on the regression plot generally, the higher the Sn-Ls/Li-Sm and Tr-Zy-Go (r, l) angles are, the lower the index values are. That is, the face is narrower when the Sn-Ls/Li-Sm and Tr-Zy-Go (r, l) angles increase. According to regression analysis, the angle Sn-Ls/Li-Sm expresses at 37% the width of the face and the angle Tr-Zy-Go (r, l) would express it at 75%.

As for the two previous curves, the face seems less large when the angle (Go.*r*-Gn-Go.*l*) is more opened; this provides 19% of the explanation related to facial shape.

Like the two previous curves, the face seems less large when the angle (GoD-Gn-GoG) is more opened. It explains at 19% the facial shape.

Angle Zy.r-Tr-Zy.l is in ordinate and indexes values of the face are in abscissa. Based on the regression chart, the more the Zy.r-Tr-Zy.l angles increase, the more the indices values grow.

However, the face gets wider when Zy.r-Tr-Zy.l angle increases. Zy.r-Tr-Zy.l angle indicates 48% of the face width.

## 4. Discussion

Many goals of physical anthropology such as studying the evolution of human body's anatomic structures, individual identification based on human remains, research of physico-racial features are all as important as analyzing the anatomic functioning of some structures like the face that plays a key role for better understanding of the evolution of human variability.

And in the current study, analysis of angles that may influence facial morphology reveals substantial trends. In order to achieve an objective analysis of the anatomic features of the facial morphology, another more detailed analysis should take into account the ethnic group or the race.

Although direct anthropometry was for years required for this aim, the use of standardized photography today is of the highest importance to achieve biological classification of individuals and for clinical research.

This is clearly noticeable through the accuracy of the face's soft tissue reproducibility minutely. Moreover, this low-cost method bears no risk of irradiation compared to cephalometry [18].

When considering measurements errors using Dahlberg's formula [17], no significant difference was noted between first and second measurements. Such matching has increased reliability of the measurements method used (Table 1). Contrary to the current study, Fortes et al. (2014) noted a significant difference between first and second measurements on the angle of the facial third, the nasolabial angle, and the lower lip/chin proportion [19]. He proved that difference is based on difficulties related to reproducibility of the points underlying these measurements.

According to the international standards (used by many authors such as Maina et al., 2012, and Yesmin et al., 2014), the faces of Ivorian subjects have been grouped from their shapes (Table 2). Therefore, 45.37% of Ivorians (comprised of 27.77% females and 17.59% men) had a wide face; 31.48% (comprised of 19.44% females and 12.03% men) had an average face; and 23.14% (comprised of 12.96% men and 10.18% females) had a narrow face [7, 8].

Through his analysis of the relationship between dental shape and facial morphotype of Ivorian women, Koffi-Gnagne et al. (2001) set a typical organization of shapes (square, diamond, round, triangular, and pear shape) that remains different from the one used in the hereby study [20].

Our results are very close to those obtained by Yesmin et al. (2014) among the Malaysians [8]. As for the Malaysian population, the facial morphology of Black Ivorians was large, average, and narrow face. Unlike most of Malaysians (45%) who had average faces, 45.37% of Ivorians had large faces, with this being the most dominant phenotype. However, it is important to specify that Yesmin et al. have not analyzed the basic reasons of that face shape variability.

Ivorian female had a wider face compared to male (80.03 ± 0.05 mm versus 83.01 ± 0.07 mm). Moreover, 27.77% of females and 17.59% of males had large faces while 19.444% of females and 12.037% of males had narrow faces (leptoprosope). This was different from results found by Farkas et al. (2005) who stated that the difference related to the average facial rate was extremely low between the two genders among subjects aged from 6 to 18 [21]. Osunwoke et al. (2011) studied sexual dimorphism bearing on facial indexes among people of Niger. They highlighted the significant difference featured by female subjects [22]. These results tightly match ours. This diversity of the face shape depends on the architectural geometry of the orofacial area. As a matter of fact and based on the analysis of different forms of face (according to the angular measurements), the wideness of the interlabial angle among the average face (122.67 ± 7.87°) and the narrow face (121.6 ± 6.15°) clearly means a retrognathia of their face (Table 5). Diouf et al. (2014) carried out a comparative study of the anatomy of the faces of Moroccans and Senegalese [23]. This value was lower than the one found among Ivorians, bearing in mind that the study made no analysis of face shapes. The middle angle Zy.*r*-Tr-Zy.*l* more opened among large face and round face is evidence of the specificity of their faces viewed in the transversal direction. Besides, the importance of zygomatic angles Tr-Zy-Go among narrow-faced individuals explains the narrowness of their faces.

**Figure 3:**
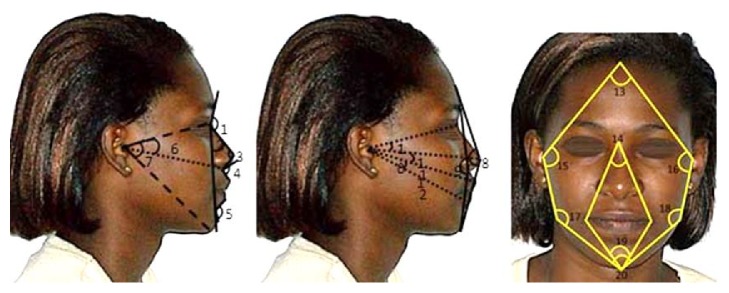
Internal and external angles of the face (full face and the profile). (1) Nasofrontal angle (G-N-Prn): the angle between the tangent lines on the nasal bridge and the glabella. (2) Nasomental angle: formed by the intersection of a line drawn between the nasion point (N), the pronasale point, and another line running from the point nasion (N) to the pogonion point (Pog). (3) Pronasale angle (N-Prn-Sn): formed by the nasion, pronasale, and subnasale points. (4) Nasolabial angle (C-Sn-Ls): it is the angle between the points columella, subnasale, and labiale superius. (5) Mentolabial angle (Li-Sm-Pog): angle formed by the lower lip, the supramental and pogonion points. (6) Angle of the middle-third of the face (N-Po-Sn): angle formed by the nasion (Sn), porion (Po), and subnasale points. (7) Angle of the lower third of the face (Sn-Po-Me): formed by the glabella (G), pronasale (Prn), and pogonion (Pog) points. (8) Facial angle (G-Prn-Pog): formed by the glabella (G), pronasale (Prn), and pogonion (Pog) points. (9) Angle of the facial convexity excluding the nose (G-Sn-Pog): formed by the intersection of the horizontal plane of Frankfort (Po-Or), a line tangential to the chin skin (Me) and to the foremost lip (L). (10) N-Po-Prn angle: formed with the nasion (Na), porion (Po), and pronasale (Prn) points. (11) Prn-Po-Ls angle: formed with the pronasale (Prn), porion (Po), and labiale superius (Ls). (12) Interlabial angle (Sn-Ls/Li-Sm): formed with the intersection of a line drawn between the subnasale point and the labiale superius and another line drawn from the lower furrow and tangent to the lower lip. (13) Zy.r-Tr-Zy.l: angle of the face width: formed by the trichion points at the two bilateral zygion points. (14) Ch.r-N-Ch.l angle: it is the nasion angle formed by the nasion point bound to the two bilateral labial commissure points. (15), (16) Left and right Tr-Zy-Go angles: left and right zygomatic angles (formed by the left and right zygion point, bound to the trichion and gonion points). (17), (18) left and right Zy-Go-Gn angles: left and right gonion angles: formed by the left and right gonion point, bound to the zygion and gnathion points at each side. (19) Angle of the oral width (Ch.r-Gn-Ch.l): formed by the gnathion point bound to the two bilateral labial commissure points. (20) Angle of the mandibular width (Go.r-Gn-Go.l) formed by the gnathion point linked to the two bilateral gonion points.* r: right; l: left*.

**Figure 4:**
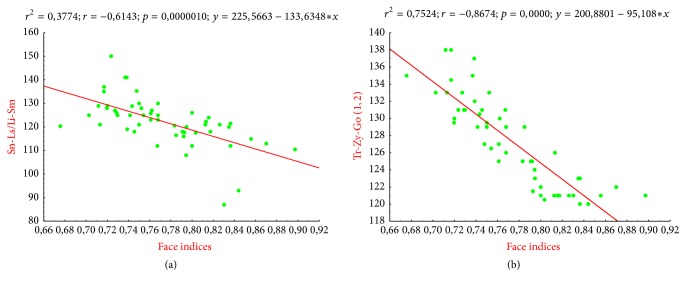
Facial index regression curve/angles Sn-Ls/Li-Sm and Tr-Zy-G.

**Figure 5:**
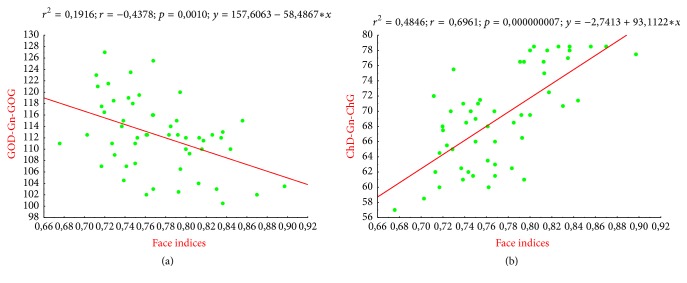
Regression curve of the facial index and Zy.r-Tr-Zy.l angle correlation.

The correlation noted between the facial indexes and the angles of the face width (Zy*.r*-Tr-Zy.*l*), interlabial (Sn-Ls/Li-Sm) angles, and the zygomatic and the mandibular width (Go.*r *** -**Gn-Go.*l*) angles means that the shape of the face is much more related to these factors (Figures 3–5, Table 6). Based on the regression line, the face is narrower when zygomatic, interlabial, and mandibular angles are more important. With linear equations defined as follows: *y* = 225,5663 − 133,6348*x*, *y* = 200,8801 − 95108*x*, and *y* = 157,6063 − 58,4867, the face width is determined by the interlabial angle in 37% of the cases, by the zygomatic angle in 75% of cases, and by the mandibular angle in 19% of cases. The angle Zy.*r*-Tr-Zy.*l* expressed the face width in about 22% of the cases with an equation defined as *y* = −0.1142 + 0.007*x*. Then the face width increases when the angle Zy.*r*-Tr-Zy.*l* also keeps increasing. Direk et al. (2016) through these anthropometric measures found a way to analyze the effect of age on facial morphology [12]. Under such form these angular measures may undoubtedly help understand some craneofacial abnormalities such as asymmetries. In this way, Bergman, 1999, in his study of the anteroposterior dysplasia of the face, has noted that acute angles should be treated with specific car in surgery [24].

Fernández-Riveiro et al. (2003) also found through their study among Galicians a nasofrontal angle of 138 ± 5.7° for males and 141 ± 9.8° for females (4). These variables were as high as those of our sample, the average of which was 131.16 ± 8.79° that implied a narrower face [25].

## 5. Conclusion

The geometrical analysis of the facial morphology of Black Ivorians subjects led to clear understanding of the differences in the forms of faces. The facial width remains tightly correlated to the bilateral (zygomatic angles) and middle angles. The more the zygomatic angles are big, the less the face is wide. The face alternatively turns significantly wide when middle angle is acute. Based on the regression, one can could predict or determine the form of face that better matches the different angular measurements. This may represent an additional factor that will help understand the morphological variability of face and also achieve therapeutic surgery planning.

Additional works taking into account both photogrammetric and direct anthropometry data will be useful for different racial groups, in the framework of a comparative study.

## Figures and Tables

**Table 1:** Description of landmarks.

Landmarks	Descriptions
Trichion (Tr)	Midpoint of the forehead where it meets the hairline
Glabella (G)	Most anterior midpoint of the forehead
Nasion (N)	Concavity in the midline at the root of the nose
Pronasale (Prn)	Tip of the nose
Columella (Cm)	Lowest point under the nose
Subnasale (Sn)	Point where the upper lip meets the columella
Labiale superius (Ls)	Point indicating the midline cutaneo-mucous border of the upper lip
Stomion (St)	Point where the closed lips meet
Labiale inferius (Li)	Point indicating the midline cutaneo-mucous border of the lower lip
Supramentale (Sm)	Deepest point of the sublabial sulcus
Pogonion (Pog)	Tip of the chin
Menton (Me)	Lowest point of the lower edge of the chin
Cervical (C)	Point where the neck meets the underside of the chin
Porion (Po)	Outermost point of the external auditory meatus
Suborbital (Or)	Palpable outer edge of the orbit
Endocanthion (En)	Inner corners of closed eyelids
Exocanthion (Ex)	Outer corners of closed eyelids
Zygion (Zy)	Most lateral points of the face, that is, outermost points of the outside contours of the zygomatic arches
Gonion (Go)	Lateral meeting points of ramus and corpus between the rearmost point of the mandibular plane and the lowest point of the ramal plane (tang ramus)
Cheilion (Ch)	Corners of the mouth
Alae (Al)	Most lateral points of the nose

**Table 2:** Facial forms (Yesmin et al., 2014).

Face shape	Range of index value
(1) Very large face	<79.9
(2) Large face	80–84.9
(3) Average or round face	85–89.9
(4) Long face or narrow face	90–94
(5) Very long face	>95

**Table 3:** Method error calculation using Dahlberg's formula.

Variable (°)	Method error
G-N-Prn	0.8
N-Prn/N-Pog	0.5
N-Prn-Sn	1
Cm-Sn- Ls	1.5
Li-Sm-Pog	0.41
N-Po-Sn	0.21
Sn-Po-Me	0.8
G-Prn-Pog	0.1
G-Sn-Pog	1.5
N-Po-Prn	0.5
Prn-Po-Ls	0.48
Sn-Ls/Li-Sn	0.1
Zy.*r*-Tr-Zy.*l*	0.75
Ch.*r*-N-Ch*.l*	0.52
Tr-Zy-Go(*r*)	0.32
Tr-Zy-Go(*l*)	0.32
Zy-Go-Gn (*r*)	0.9
Zy-Go-Gn (*l*)	0.9
Ch.*r*-Gn-Ch.*l*	0.6
Go.*r* -Gn-Go.*l*	0.22

**Table 4:** Distribution of facial shapes based on gender.

Sexes	Facial indices	Large face 80–85	Average face 85–90	Long face 90–95	Total	*p*
Male	83.01 ± 0.07	19	13	14	46	0.03
Female	80.03 ± 0.05	30	21	11	62	

Total	81.52 ± 0.06	49	34	25	108	

%		45.37%	31.48%	23.15%		

*p* < 0.05 significant;  *p* > 0.5 not significant (NS); SD: standard deviation.

**Table 5:** Comparison of angular variables according to facial shapes.

Facial forms	Large face (*n* = 49)	Average face (*n* = 34)	Long face (*n* = 25)	*p*
Parameters	Mean ± SD	Mean ± SD	Mean ± SD	
G-N-Prn	129.4 ± 9.89	131.01 ± 8.7	133.00 ± 7.8	NS
N-Prn/N-Pog	34.31 ± 1.21	34.61 ± 1.4	32.00 ± 3.78	NS
N-Prn-Sn	96.47 ± 3.17	97.03 ± 2.13	97.12 ± 6.16	NS
Cm-Sn-Ls	88.0 ± 5.01	89.97 ± 6.21	88.76 ± 5.33	NS
Li-Sm-Pog	117.21 ± 19.7	117.44 ± 17.81	116.53 ± 17.33	NS
N-Po-Sn	25.63 ± 2.11	26.02 ± 2.99	25.64 ± 2.32	NS
Sn-Po-Me	41.87 ± 7.51	40.85 ± 6.8	39.75 ± 7.02	NS
G-Prn-Pog	145.9 ± 3.23	143.87 ± 2.31	146.65 ± 4.33	NS
G-Sn-Pog	163.1 ± 4.61	164.17 ± 5.3	166.31 ± 6.02	NS
N-Po-Prn	19.78 ± 2.47	18.99 ± 3.72	18.79 ± 3.17	NS
Prn-Po-Ls	13.02 ± 1.42	12.25 ± 1.63	12 ± 1.71	NS
Sn-Ls/Li-Sn	110.78 ± 7.42^*∗∗*^	122.67 ± 7.87^*∗*^	121.6 ± 6.15^*∗*^	<0.01
Zy.*r*-Tr-Zy.*l*	87.89 ± 6.58^*∗*^	86.17 ± 4.73^*∗*^	81.8 ± 9.57^*∗∗*^	<0.001
Chy*.r*-N-Chy.*l*	47.98 ± 2.51	49.23 ± 3.1	48.35 ± 3.22	NS
Tr-Zy-Go *(r*;* l)*	125.31 ± 1.4^*∗*^	125.75 ± 2.13^*∗*^	131.67 ± 1.5^*∗∗*^	<0.004
Zy-Go-Gn *(r*;* l)*	131.65 ± 2.71	133.31 ± 2.79	133.72 ± 3.8	NS
Ch.*r*-Gn-Ch.*l*	67.21 ± 3.12	65.2 ± 4.4	64.23 ± 6.3	NS
Go.*r*-Gn-Go.*l*	114.87 ± 4.51^*∗*^	114.5 ± 4.71^*∗*^	107.63 ± 4.27^*∗∗*^	<0.04

^*∗*^ *p* < 0.05 significant; *p* > 0.5 not significant (NS). SD: standard deviation; Bonferroni test for the grouping of subjects in *∗*, *∗∗*. *r*: right; *l*: left.

**Table 6:** Correlation between indices values and angles measurements of the face.

Values of face			
Parameters	Mean ± SD	CC	*p*
G-N-Prn	131.16 ± 8.79	−,0520	NS
N-Prn/N-Pog	33.67 ± 2.38	−,0940	NS
N-Prn-Sn	96.87 ± 4.7	,0673	NS
Cm-Sn- Ls	88.91 ± 5.8	,0079	NS
Li-Sm-Pog	117.06 ± 18.18	−,1077	NS
N-Po-Sn	25.76 ± 2.23	,0764	NS
Sn-Po-Me	40.82 ± 7.82	−,0769	NS
G-Prn-Pog	145.47 ± 3.6	−,0321	NS
G-Sn-Pog	164.52 ± 5.4	,0779	NS
N-Po-Prn	19.18 ± 3.59	−,1268	NS
Prn-Po-Ls	12.45 ± 1.5	−,2251	NS
Sn-Ls/Li-Sn	118.35 ± 7.52	−,6143	0.01
ZyD-Tr-ZyG	85.28 ± 6.77	,6961	0.00
ChyD-N-ChyG	48.52 ± 2	−,2122	NS
Tr-Zy-Go *(r*;* l)*	127.57 ± 1.89	−,8674	0,00
Zy-Go-Gn *(r*;* l)*	132.89 ± 3.02	−,0696	NS
Ch*r*-Gn-Ch*l*	65.54 ± 5	−,1401	NS
Go*r*-Gn-Go*l*	112.3 ± 4.5	−,4378	0.04

NS: not significant; SD: standard deviation: *p*: significant; CC: correlation coefficient.
